# Fatigue and cognitive impairment after COVID-19: A prospective multicentre study

**DOI:** 10.1016/j.eclinm.2022.101651

**Published:** 2022-09-17

**Authors:** Tim J. Hartung, Christian Neumann, Thomas Bahmer, Irina Chaplinskaya-Sobol, Matthias Endres, Johanna Geritz, Karl Georg Haeusler, Peter U. Heuschmann, Hanna Hildesheim, Andreas Hinz, Sina M. Pütz, Anna Horn, Michael Krawczak, Lilian Krist, Jennifer Kudelka, Wolfgang Lieb, Corina Maetzler, Anja Mehnert-Theuerkauf, Felipe A. Montellano, Caroline Morbach, Sein Schmidt, Stefan Schreiber, Flo Steigerwald, Stefan Störk, Walter Maetzler, Carsten Finke

**Affiliations:** a Klinik und Hochschulambulanz für Neurologie, Charité-Universitätsmedizin Berlin, Berlin, Germany; b Department of Medical Psychology and Medical Sociology, University Medical Center Leipzig, Leipzig, Germany; c Neurology Department, University Medical Center Schleswig-Holstein, Campus Kiel, Kiel, Germany; d Internal Medicine Department I, University Hospital Schleswig Holstein, Campus Kiel, Kiel, Germany; e Airway Research Center North (ARCN), German Center for Lung Research (DZL), Grosshansdorf, Germany; f Department of Medical Informatics, University Medical Center Göttingen, Göttingen, Germany; g Center for Stroke Research Berlin, Berlin, Germany; h Excellence Cluster NeuroCure, Berlin, Germany; i German Center for Neurodegenerative Diseases (DZNE), Partner Site Berlin, Germany; j German Centre for Cardiovascular Research (DZHK), Partner Site Berlin, Germany; k Department of Neurology, Universitätsklinikum Würzburg, Würzburg, Germany; l Institute of Clinical Epidemiology and Biometry, University of Würzburg, Würzburg, Germany; m Clinical Trial Center, University Hospital Würzburg, Würzburg, Germany; n University of Cologne, Faculty of Medicine and University Hospital Cologne, Department I of Internal Medicine, Center for Integrated Oncology Aachen Bonn Cologne, Duesseldorf, Germany; o Institute of Medical Informatics and Statistics, Kiel University, University Medical Center Schleswig-Holstein Campus Kiel, Kiel, Germany; p Institute of Social Medicine, Epidemiology and Health Economics, Charité-Universitätsmedizin Berlin, Berlin, Germany; q Institute of Epidemiology, Kiel University, Kiel, Germany; r Comprehensive Heart Failure Center, University and University Hospital Würzburg, Würzburg, Germany; s University Hospital Würzburg, Department for Medicine I and Comprehensive Heart Failure Center, Würzburg, Germany; t Berlin Institute of Health at Charité – Universitätsmedizin Berlin, Clinical Study Center, Berlin, Germany; u Universitätsklinikum Schleswig-Holstein, Christian-Albrechts-Universität zu Kiel, Klinik für Innere Medizin I, Kiel, Germany; v Department of Clinical Research & Epidemiology, Comprehensive Heart Failure Center, University Hospital Würzburg, Würzburg, Germany; w Department of Internal Medicine I, University Hospital Würzburg, Würzburg, Germany

**Keywords:** COVID-19, SARS-CoV-2, Post-acute COVID-19 syndrome, Fatigue, Cognitive dysfunction

## Abstract

**Background:**

Reliable estimates of frequency, severity and associated factors of both fatigue and cognitive impairment after COVID-19 are needed. Also, it is not clear whether the two are distinct sequelae of COVID-19 or part of the same syndrome.

**Methods:**

In this prospective multicentre study, frequency of post-COVID fatigue and cognitive impairment were assessed in *n* = 969 patients (535 [55%] female) ≥6 months after SARS-CoV-2 infection with the FACIT-Fatigue scale (cut-off ≤30) and Montreal Cognitive Assessment (≤25 mild, ≤17 moderate impairment) between November 15, 2020 and September 29, 2021 at University Medical Center Schleswig-Holstein, Campus Kiel and University Hospital Würzburg in Germany. 969 matched non-COVID controls were drawn from a pre-pandemic, randomised, Germany-wide population survey which also included the FACIT-Fatigue scale. Associated sociodemographic, comorbid, clinical, psychosocial factors and laboratory markers were identified with univariate and multivariable linear regression models.

**Findings:**

On average 9 months after infection, 19% of patients had clinically relevant fatigue, compared to 8% of matched non-COVID controls (*p* < 0.001). Factors associated with fatigue were female gender, younger age, history of depression and the number of acute COVID symptoms. Among acute COVID symptoms, altered consciousness, dizziness and myalgia were most strongly associated with long-term fatigue. Moreover, 26% of patients had mild and 1% had moderate cognitive impairment. Factors associated with cognitive impairment were older age, male gender, shorter education and a history of neuropsychiatric disease. There was no significant correlation between fatigue and cognitive impairment and only 5% of patients suffered from both conditions.

**Interpretation:**

Fatigue and cognitive impairment are two common, but distinct sequelae of COVID-19 with potentially separate pathophysiological pathways.

**Funding:**

German Federal Ministry of Education and Research (BMBF).

**Research in context**

**Evidence before this study**

A literature search on PubMed of articles published between 1 March 2020 and 19 January 2022 using the search terms “fatigue” and “cognitive impairment” as well as “LitCLONGCOVID [filter]” was conducted before data analysis. A meta-analysis by Ceban and colleagues estimated that the proportion of individuals experiencing fatigue 12 or more weeks following COVID-19 diagnosis was 0.32 and the proportion of individuals exhibiting cognitive impairment was 0.22. Since these estimates were derived from studies without control groups and with a variety of instruments, it was unclear whether these rates were higher or lower than in individuals without COVID. Research on associated factors of post-COVID fatigue and cognitive impairment was inconclusive.

**Added value of this study**

By assessing fatigue with a validated instrument in both a representative sample of patients with COVID and in well-matched non-COVID controls, we are able to show that the rate of fatigue is indeed higher ≥6 months after SARS-CoV-2 infection. In addition, we show that fatigue and cognitive impairment affect different age groups and differ in their associated factors.

**Implications of all the available evidence**

Rates of fatigue are substantially and statistically significantly elevated in patients after SARS-CoV-2 infection. Fatigue and cognitive impairment are distinct sequelae of COVID-19 and may arise from different pathophysiological pathways.

Alt-text: Unlabelled box

## Introduction

Post-COVID syndrome is defined as symptoms that develop during or after COVID-19 and last longer than 12 weeks.^1^ Fatigue is the most frequently reported post-COVID symptom and represents a major cause of disability and reduced quality of life.^2^, ^3^, ^4^ As such, post-COVID fatigue is a public health challenge potentially affecting millions of patients world-wide.^5^ Accurate estimates of the frequency and severity of post-COVID fatigue are required to inform public health measures,^3^ but previous studies included only relatively few patients, did not use validated instruments or lacked adequate non-COVID control groups.^6^ A recent meta-analysis of data obtained with different fatigue measures concluded that one third of COVID-19 patients are affected by persisting fatigue.^6^ However, it is not clear how this proportion compares to the prevalence of fatigue in the general population, estimates of which range from 1% to well over 50% depending on the method of assessment.^7^^,^^8^ To resolve this uncertainty, studies are urgently needed that assess fatigue in representative groups of patients with COVID-19 and non-COVID controls, using the same validated instruments.^9^^,^^10^

Furthermore, it is essential to identify determinants of persisting fatigue to inform research on underlying mechanisms and to develop efficient health service procedures.^9^ However, so far, only anxiety and depression have been consistently found to be associated with post-COVID fatigue,^11^, ^12^, ^13^ while research on other associated factors such as gender, aspects of initial disease severity and inflammatory markers remained inconclusive.^6^^,^^13^ Moreover, about 20% of patients have post-COVID cognitive impairment,^6^ which is one of the most debilitating aspects of post-COVID syndrome.^14^ Currently, it is still unclear whether fatigue and cognitive impairment are distinct sequelae of COVID-19 or may be part of the same syndrome sharing the same set of risk factors and underlying pathophysiology. In part, this is due to the fact that most studies included small sample sizes, lacking power to adequately control for sociodemographic, clinical and psychosocial covariates.^6^ Larger studies covering a wide range of potential covariates are hence required to identify independent predictors of fatigue and cognitive impairment.

Here, we prospectively estimated the frequency of fatigue in a large, population-based sample of COVID-19 patients ≥6 months after SARS-CoV-2 infection, compared to gender- and age-matched non-COVID controls. In addition, we estimated the frequency of cognitive impairment ≥6 months after infection and assessed sociodemographic, comorbid, clinical and psychosocial factors as well as laboratory markers associated with fatigue and cognitive impairment.

## Methods

### Participants

COVIDOM is a population-based, prospective multicentre study in the German National Pandemic Cohort Network (NAPKON).^15^^,^^16^ Patient inclusion criteria were: (i) a positive polymerase chain reaction (PCR) test for SARS-CoV-2, (ii) a primary residence in in the administrative regions of Schleswig-Holstein or Würzburg, and (iii) age ≥18 years at the time of infection. Patients were invited by mail through public health authorities. Exclusion criteria comprised: (i) less than 6 months between SARS-CoV-2 infection and study visit and (ii) acute reinfection with SARS-CoV-2 at the time of the scheduled study visit. Further details on design, methods and overall sample size calculation are provided the study protocol.^17^ Patients were assessed between November 15, 2020 and September 29, 2021 at University Medical Center Schleswig-Holstein, Campus Kiel and University Hospital Würzburg in Germany.

The present analysis required a minimal sample size of *N* = 1000 to allow for meaningful subgroup analyses (e.g. by age group), based on prior effect-size estimates.^15^, ^17^ Accordingly, 1000 cases with complete data on the Montreal Cognitive Assessment (MoCA) and the FACIT-Fatigue questionnaire were randomly selected. To rule out non-wild-type variants of SARS-CoV-2, this analysis includes only participants with initial PCR before February 14, 2021.

Data from a representative population survey assessing fatigue in 2576 participants were used to select a COVID-free control group.^18^ This survey randomly selected participants from the Germany-wide resident registers and assessed fatigue using the FACIT-Fatigue scale during home visits between March and May 2015. Controls were matched to cases by gender and age using R package *e1071*.

### Measures

Fatigue was assessed using the 13-item FACIT fatigue scale, a widely-used and validated self-report questionnaire to assess symptoms on a five-point Likert-scale with a sum score ranging from 0 (worst fatigue) to 52 (no fatigue).^18^, ^19^, ^20^ Clinically relevant fatigue was defined by scores ≤30, as suggested by the creators of the scale, based on general population data.^21^

The MoCA was used to assess cognitive performance in patients. The MoCA is an established and validated screening instrument yielding a total score between 0 (most severe cognitive impairment) and 30 (no cognitive impairment).^22^ Following the test manual, one extra point was added to scores from individuals with fewer than 12 years of education. Scores ≥26 were interpreted as normal, 18–25 mild, 10–17 moderate and ≤9 as severe cognitive impairment.

Sociodemographic and clinical characteristics of cases were collected through standardised self-report questionnaires. At in-person appointments, cognitive tests, psychosocial assessments and a physical examination were performed, and blood samples taken. Information on pre-COVID comorbidity and 22 typical acute COVID symptoms (Supplementary Materials, eTable 1) was collected in a standardised clinical interview and collated with medical records.

Depressive symptoms were evaluated with the PHQ-8 questionnaire,^23^ anxiety with the GAD-7^24^ and sleep disturbances with the Pittsburgh Sleep Quality Index (PSQI).^25^ Anaemia was defined as Hb < 12.0 g/dl in women and < 13.6 g/dl in men. The cut-off for C-reactive protein (CRP) elevation was ≥5 mg/l.

### Statistical analysis

Statistical tests were performed in R version 4.0.2, two-tailed and p-values < 0.05 were considered statistically significant. All figures were plotted using R package ggplot2.

FACIT and MoCA scores were roughly normally distributed, but with a ceiling effect. Since Shapiro-Wilk tests indicated non-normality for both variables, Wilcoxon rank-sum tests were used for group comparisons. Effect sizes were quantified as Hedges’ g. Frequencies of fatigue in cases and controls were compared using chi-squared tests.

To evaluate associated factors of fatigue and cognitive impairment, we created two models for each of the two outcomes. Model 1 contained potential predictors assessed during the acute stage of COVID-19 as independent variables, i.e. gender, age, education, pre-COVID comorbidity, number of acute COVID symptoms and COVID treatment setting. Model 2 contained concomitant factors during the post-COVID period as independent variables, i.e. gender, age, education, time since COVID diagnosis, laboratory confirmed anaemia, CRP, depressive symptoms, anxiety symptoms, sleep problems, and respectively MoCA or FACIT score.

Potential associated factors were first studied in univariate linear regression models. Then, we created multivariable linear regression models for all possible subsets of potential associated factors using the *leaps* package version 3.1. Models were compared using Mallow's Cp, which assesses model fit with a penalty for increased model complexity (i.e., the number included parameters). Since lower Cp values indicate a better model fit, the model with the lowest Cp was chosen as the final model. Multicollinearity was assessed using the Variance Inflation Factor (VIF) and homoscedasticity was visually assessed with Q-Q plots.

There were ≤5% missing values for all analysed variables, except PSQI (*n* = 634, 35% missing). Missing values were deleted in pairwise fashion, if possible, and case-wise fashion otherwise. Removing the PSQI from the regression models did not result in relevant changes in model fit or parameter selection.

### Ethics and study registration

All patients and healthy control participants provided written informed consent to the respective studies. Both studies were approved by the responsible ethics committees (COVIDOM reference numbers: Kiel D537/20, Berlin EA1/316/21). The COVIDOM study is registered at the German registry for clinical studies (DRKS00023742) and at ClinicalTrials.gov (NCT04679584).

### Role of the funding source

The funders were not involved in study design, data collection, data analysis, interpretation of data, writing of the report or decision to submit the paper for publication. TJH, CN, WM, and CF had access to the data and are finally responsible for the decision to submit the current work for publication.

## Results

### Participants

At the time of data analysis, 1812 (35%) out of 5133 eligible patients with COVID-19 had agreed to participate in our study (Supplementary Materials, eFigure 1). After randomized selection of 1000 potential participants, 31 cases were excluded due to missing data or PCR test dates outside the study period, leading to a final sample size of *N* = 969 cases.

Sample characteristics are shown in Table 1. Median duration between infection and study visit was 9 months (interquartile range 8 to 12 months). The most common pre-COVID comorbidities were cardiovascular, especially arterial hypertension (*n* = 222, 23%), or neuropsychiatric, especially depression (*n* = 102, 11%) and migraine (*n* = 93, 10%). None of the patients had a dementia diagnosis. Details on pre-COVID comorbidity and blood tests are shown in the Supplementary Materials, eTable 1.

**Table 1:** Sample characteristics of patients after SARS-CoV-2 infection (*n* = 969) and matched non-COVID controls (*n* = 969).

Characteristic	Patients	Controls	*p*
**Sociodemographic**			
Gender			>0.999
Female	535 (55%)	535 (55%)	
Male	434 (45%)	434 (45%)	
Age [years]			0.995
18–34	278 (29%)	278 (29%)	
35–49	204 (21%)	204 (21%)	
50–64	374 (39%)	374 (39%)	
65–87	113 (12%)	113 (12%)	
School education			< 0.001
< 12 years	458 (49%)	761 (79%)	
≥ 12 years	485 (51%)	207 (21%)	
**Clinical characteristics**			
Pre-COVID comorbidity			
Any neuropsychiatric disease	246 (26%)		
Cardiovascular disease	282 (29%)		
Sleep apnoea	44 (5%)		
Tumour disease	15 (2%)		
Chronic kidney disease	4 (1%)		
Time since SARS-CoV-2 infection			
6–9 months	470 (49%)		
9–12 months	344 (36%)		
≥12 months	155 (16%)		
Treatment setting			
Home isolation	908 (94%)		
General ward	46 (5%)		
Intensive care	15 (2%)		
Number of acute COVID symptoms			
Asymptomatic	48 (5.1%)		
1–5	265 (28%)		
6–8	231 (24%)		
9–11	219 (23%)		
12–21	186 (20%)		
Anaemia	74 (8%)		
C-reactive protein elevation (≥5 mg/l)	77 (8%)		
Depression symptom severity (PHQ-8)			
Minimal	523 (55%)		
Mild	273 (29%)		
Moderate	103 (11%)		
Moderately Severe	38 (4%)		
Severe	9 (1%)		
Anxiety symptom severity (GAD-7)			
Minimal	685 (72%)		
Mild	180 (19%)		
Moderate	59 (6%)		
Severe	24 (3%)		
Sleep disturbance (PSQI ≥5)	513 (81%)		

In the 2015 general population survey, 2576 out of 4844 (53%) individuals participated. Cases and selected controls were well matched by gender and age (Table 1). Additional matching by education had no relevant effects on the main results.

### Frequency of fatigue and cognitive impairment

**Figure 1:**
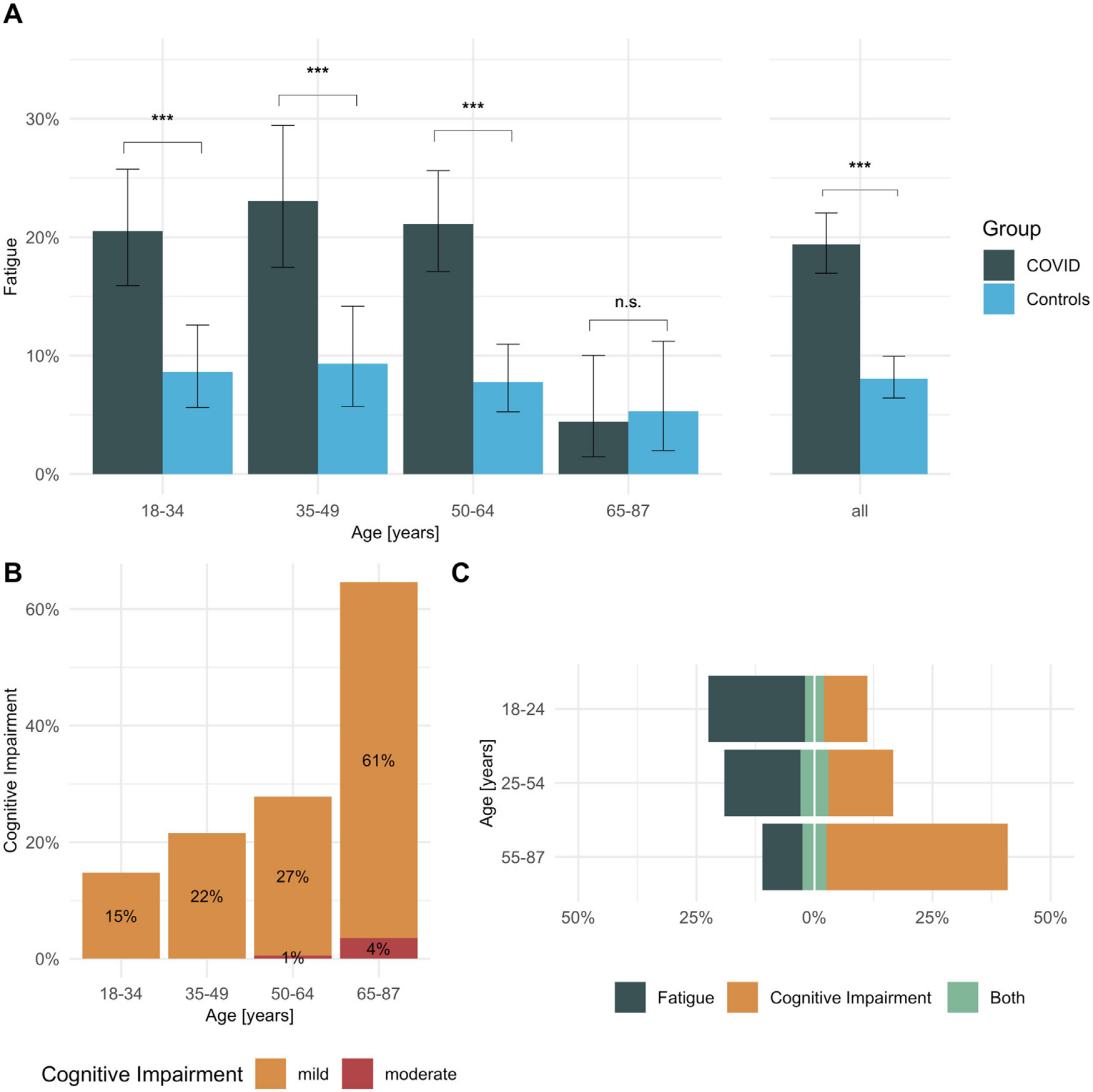
(A) Frequency of fatigue by age in patients ≥6 months after SARS-CoV-2 infection (*n* = 969) and matched general population controls (*n* = 969); (B) frequency of mild and moderate cognitive impairment in *n* = 969 patients by age; (C) Fatigue was more common in younger patients, compared to cognitive impairment, which was more common in older patients. Between 4% and 6% of patients suffered from both fatigue and cognitive impairment. Error bars indicate 95% confidence intervals, *** *p* < 0.001, n.s. non-significant.

On average 9 months after infection, patients had statistically significantly lower FACIT scores than matched non-COVID controls (mean ± SD, 39.2 ± 10.5 vs. 43.6 ± 8.5; Wilcoxon W=337812, *p* < 0.001) with a medium effect size (Hedges’ g=-0.46, 95%-confidence interval (CI): [-0.55, -0.37]). Overall, 188 of 969 cases (19%, 95%-CI: [17%, 22%]) and 78 of 969 controls (8%, 95%-CI: [6%, 10%]) had FACIT scores indicating clinically relevant fatigue (χ^2^=51. 771, *p* < 0.001). Fatigue was statistically significantly more common in cases than controls in all age groups except >65 years (Figure 1A).

Overall, 256 of 969 cases (26%) had MoCA scores indicating mild and 7 of 969 (1%) moderate cognitive impairment, both of which were more common in older age groups than in younger ones (Figure 1B). While the relative frequency of fatigue was highest in patients younger than 25 years and decreased with age, the occurrence of cognitive impairment increased with age (Figure 1C). Overall, only 53 of 969 (5%) patients suffered from both cognitive impairment and fatigue, compared to 135 of 969 (14%) with isolated fatigue, and 210 of 969 (22%) with isolated cognitive impairment.

### Factors associated with fatigue

Univariate associations with FACIT fatigue scores are shown in Table 2. The best-fitting multivariable predictor model (Model 1) contained 6 variables, of which female gender, younger age, pre-COVID neuropsychiatric comorbidity, pre-COVID depression, and the number of acute COVID-19 symptoms remained as statistically significant predictors of persisting fatigue (R^2^=0.21, *p* < 0.001; Table 2). There were no signs of multicollinearity (VIF range: 1.04 to 1.55) and Q-Q plots showed no sign of relevant heteroscedasticity.

**Table 2:** Linear regression models for univariate associations, predictors and concomitant factors of FACIT fatigue scores.

		Univariate				Model 1: Predictors (*n* = 907)				Model 2: Concomitant factors (*n* = 615)			
	Characteristic	B	95% CI	Std. beta	*p*	B	95% CI	Std. beta	*p*	B	95% CI	Std. beta	*p*
Sociodemographic	Gender				**< 0.001**			0.11	**< 0.001**				
	Female	—	—			—	—						
	Male	3.48	2.17, 4.80	0.17		2.37	1.11, 3.63						
	Age [years]	0.07	0.03, 0.11	0.11	**< 0.001**	0.08	0.04, 0.12	0.11	**< 0.001**				
	Education				0.683								
	< 12 years	—	—										
	≥12 years	−0.28	−1.62, 1.06	−0.01									
Potential predictors	Any neuropsychiatric disease	−5.76	−7.23, −4.28	−0.24	**< 0.001**	−1.98	−3.72, −0.24	−0.08	**0.026**				
	Depression disorder	−8.72	−10.82, −6.62	−0.26	**< 0.001**	−5.03	−7.51, −2.56	−0.15	**< 0.001**				
	Anxiety disorder	−5.21	−8.81, −1.61	−0.09	**0.005**								
	Sleep apnoea	−3.34	−6.51, −0.18	−0.07	**0.038**	−2.65	−5.65, 0.34	−0.05	0.082				
	Chronic kidney disease	0.16	−5.56, 5.88	0.00	0.956								
	Cardiovascular disease	0.21	−1.25, 1.68	0.01	0.773								
	Tumour disease	−1.79	−7.14, 3.56	−0.02	0.511								
	Number of acute COVID symptoms	−0.87	−1.01, −0.73	−0.36	**< 0.001**	−0.76	−0.91, −0.62	−0.32	**< 0.001**				
	Treatment setting				0.777								
	Home isolation	—	—	0.02									
	General ward	−0.33	−3.44, 2.78	−0.01									
	Intensive care	−1.87	−7.23, 3.49	−0.02									
Concomitant factors	Time since diagnosis [days]	0.03	0.02, 0.04	0.16	**< 0.001**					0.01	0.00, 0.01	0.04	0.062
	Anaemia	1.39	−1.10, 3.88	0.04	0.274								
	C−reactive protein [mg/l]	−0.25	−0.45, −0.05	−0.08	**0.013**								
	Cognition (MoCA)	−0.04	−0.30, 0.23	−0.01	0.784								
	Depression (PHQ-8)	−1.95	−2.03, −1.86	−0.83	**< 0.001**					−1.61	−1.74, −1.47	−0.69	**< 0.001**
	Anxiety (GAD-7)	−1.78	−1.91, −1.65	−0.66	**< 0.001**								
	Sleep (PSQI)	−1.71	−1.86, −1.55	−0.65	**< 0.001**					−0.51	−0.66, −0.35	−0.19	**< 0.001**

The best-fitting post-hoc model for acute COVID-19 symptoms predicting post-COVID fatigue contained 7 out of 22 assessed symptoms (altered consciousness, dizziness, myalgia, thorax pain, dyspnoea, dysosmia and rash), all of which showed statistically significant associations in the multivariable model (Supplementary Materials, eTable 2). Among these, altered consciousness (β=-4.1), dizziness (β=-3.1) and myalgia (β=-2.7) showed the strongest associations (all *p* < 0.001).

The best-fitting multivariable model of concomitant factors associated with fatigue (Model 2) only contained time since COVID-19 diagnosis, depressive symptoms and sleep problems, of which the latter two remained statistically significant (R^2^=0.70, *p* < 0.001, VIF range: 1.01 to 1.81, no heteroscedasticity in Q-Q plots; Table 2).

### Factors associated with cognitive impairment

Univariate associations with MoCA scores are shown in Table 3. The best fitting multivariable regression model for predictors of cognitive impairment (Model 1) contained 8 variables, out of which male gender, older age, shorter education, and neuropsychiatric comorbidity remained as statistically significant predictors (R^2^=0.21, *p* < 0.001, VIF range: 1.05 to 1.68, no heteroscedasticity in Q-Q plots; Table 3). In this model, there was no statistically significant association between measures of COVID-19 disease severity and cognitive impairment after adjusting for sociodemographic characteristics and pre-COVID comorbidity.

**Table 3:** Linear regression models for univariate associations, predictors and concomitant factors of Montreal Cognitive Assessment total score.

		Univariate				Model 1: Predictors(*n* = 913)				Model 2: Concomitant factors(*n* = 922)			
	Characteristic	B	95% CI^1^	Std. beta	*p*	B	95% CI^1^	Std. beta	*p*	B	95% CI^1^	Std. beta	*p*
Sociodemographic	Gender				0.050			-0.07	**0.030**			-0.07	**0.024**
	Female	—	—			—	—			—	—		
	Male	−0.31	−0.63, 0.00	−0.06		−0.33	−0.63, −0.03			−0.34	−0.64, −0.05		
	Age [years]	−0.06	−0.07, −0.05	−0.37	**< 0.001**	−0.04	−0.05, −0.03	−0.25	**< 0.001**	−0.05	−0.06, −0.04	−0.31	**< 0.001**
	Education				**< 0.001**			0.23	**< 0.001**			0.22	**< 0.001**
	< 12 years	—	—			—	—			—	—		
	≥12 years	1.5	1.2, 1.8	0.31		1.1	0.82, 1.4			1.1	0.80, 1.4		
Potential predictors	Any neuropsychiatric disease	−0.75	−1.1, −0.39	−0.13	**< 0.001**	−0.80	−1.2, −0.37	−0.14	**< 0.001**				
	Depression disorder	−0.26	−0.77, 0.26	−0.03	0.33	0.53	−0.05, 1.1	0.07	0.074				
	Anxiety disorder	0.30	−0.57, 1.2	0.02	0.50	0.67	−0.18, 1.5	0.05	0.12				
	Sleep apnoea	−1.1	−1.9, −0.39	−0.10	**0.003**								
	Chronic kidney disease	−0.56	−1.9, 0.79	−0.03	0.41								
	Cardiovascular disease	−1.3	−1.6, −1.0	−0.24	**< 0.001**	−0.29	−0.66, 0.07	−0.05	0.12				
	Tumour disease	−1.0	−2.3, 0.24	−0.05	0.11								
	Number of acute COVID symptoms	−0.02	−0.06, 0.01	−0.04	**< 0.001**								
	Treatment setting				**< 0.001**				0.15				
	Home isolation	—	—	0.17		—	—	0.08					
	General ward	−1.6	−2.4, −0.91	−0.14		−0.54	−1.2, 0.16	0.02					
	Intensive care	−2.0	−3.2, −0.74	−0.10		−0.80	−2.0, 0.37	−0.02					
Concomitant factors	Time since diagnosis [days]	0.00	−0.01, 0.00	−0.11	**< 0.001**								
	Anaemia	−0.30	−0.88, 0.28	−0.03	0.31								
	C-reactive protein [mg/l]	0.01	−0.04, 0.05	0.01	0.76								
	Fatigue (FACIT)	0.00	−0.02, 0.01	−0.01	0.78					0.00	−0.02, 0.02	0.01	0.7
	Depression (PHQ-8)	0.00	−0.03, 0.04	0.00	0.94								
	Anxiety (GAD-7)	−0.01	−0.05, 0.03	−0.01	0.78					−0.03	−0.08, 0.02	−0.04	0.3
	Sleep (PSQI)	−0.06	−0.10, −0.02	−0.11	**0.004**								

In the best-fitting multivariable model of concomitant factors associated with cognitive impairment (Model 2), which contained 5 variables, only sociodemographic characteristics showed statistically significant associations (R^2^=0.19, VIF range: 1.01 to 1.81, no heteroscedasticity in Q-Q plots; Table 3).

### Time since diagnosis

**Figure 2:**
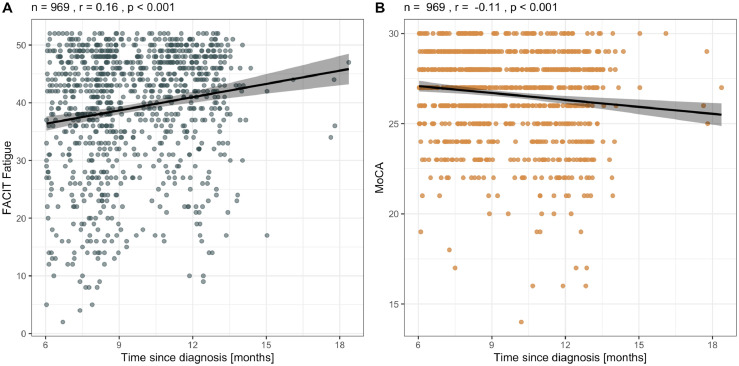
Association between time since diagnosis of SARS-CoV-2 infection and (A) FACIT fatigue score and (B) Montreal Cognitive Assessment (MoCA) score. r, Pearson correlation coefficient, trend line indicates univariate linear regression, shaded area 95% confidence interval.

Time since SARS-CoV-2 infection showed a small negative correlation with fatigue severity (FACIT: *r* = 0.16, *p* < 0.001) and a small positive correlation with severity of cognitive impairment (MoCA: *r* = -0.11, *p* < 0.001; Figure 2). Since only seven patients were assessed > 15 months after infection and could be considered outliers in this regard, we repeated the analysis excluding these cases and found no relevant change to the results (FACIT: *r* = 0.17, *p* < 0.001; MoCA: *r* = -0.13, *p* < 0.001; Supplementary Materials, eFigure 2).

## Discussion

In this prospective multicentre study including 969 non-hospitalised and hospitalised patients with COVID-19 and 969 matched controls, we found that 19% of patients had clinically relevant levels of fatigue at a median of 9 months after SARS-CoV-2 infection, compared to 8% of controls who were assessed with the same validated instrument. Younger patients were more severely affected than older patients and there was a negative association between the time since SARS-CoV-2 infection and fatigue severity, suggesting that post-COVID fatigue may improve over time. Importantly, only 5% of patients had both fatigue and cognitive impairment and there was no significant association between the two syndromes.

Patients had a more than two-fold higher rate of fatigue compared to matched controls in our analysis. This frequency is lower than the estimated 32% in a recent meta-analysis^6^; however, studies included in this meta-analysis were heterogeneous with respect to fatigue instruments and sample size. Considering that the number of global SARS-CoV-2 infections recently exceeded 500 million, many million people world-wide will suffer from fatigue-related reduced quality of life,^2^ impaired daily life functions and may not be able to return to work because of fatigue.^6^ Importantly, patients with post-COVID fatigue will require professional help, e.g. through specialized interdisciplinary outpatient clinics and rehabilitation programs.

The best predictor of post-COVID fatigue was the number of acute COVID symptoms. Among these, altered consciousness, dizziness and myalgia showed the strongest association. In contrast, COVID disease severity (in terms of home isolation vs. general hospital ward vs. intensive care) was not significantly associated with long-term fatigue, which is consistent with findings from a recent meta-analysis and a large cohort study in hospitalised patients.^6^^,^^26^ This suggests that the affected organ systems, especially central and peripheral nervous system involvement, are more relevant for the development of fatigue than overall illness severity.^27^

The pathophysiological mechanisms underlying post-COVID fatigue remain largely elusive. However, accumulatingevidence points to a virus-related autoimmune aetiology.^28^ Both fatigue and increased autoimmunity are common sequela of infections with SARS-CoV-1, MERS, Epstein-Barr, herpes and hepatitis viruses^29^, ^30^ and fatigue is a common symptom of autoimmune diseases of the central nervous system such as multiple sclerosis^31^ and autoimmune encephalitis.^32^, ^33^ Indeed, acute patients with COVID-19 who have neurological symptoms also show high frequencies of CSF autoantibodies.^34^ Furthermore, measures of inflammation have been found to be associated with overall post-COVID syndrome severity, independent of confounding inflammatory comorbidities, and COVID-related mortality, and have thus been suggested as diagnostic and prognostic biomarkers.^35^^,^^36^ Indeed, C-reactive protein (CRP) levels were significantly correlated with concomitant fatigue severity in our univariate linear regression analysis (standardised beta=-0.08, *p* = 0.013), but showed no association in multivariable models. Future studies should investigate a wider spectrum of pro-inflammatory cytokines as potential biomarkers of post-COVID fatigue.

Recent imaging and pathology studies point to persistent brain damage in patients with post-COVID syndrome and non-human primates infected with SARS-CoV-2.^37^, ^38^ Moreover, microstructural damage of the basal ganglia was recently shown to be associated with fatigue severity in patients with post-COVID syndrome, linking evidence of basal ganglia involvement in MS-related fatigue and post-COVID fatigue.^39^ In the context of these findings, our results suggest that early neurological involvement in COVID-19 may pave the way for long-term neuropsychiatric morbidity.

Of note, younger patients reported more severe fatigue than older patients in our study, while previous studies in COVID-19 patients have shown heterogeneous results in this regard^6^ and no strong age effects have been found in the general population.^18^ It is possible that working-aged patients may be particularly impaired by fatigue, especially if they are unable to return to work, and thus might experience fatigue as more severe than retired patients. Alternatively, immunological age or hormonal factors may play a relevant role in the pathophysiology of fatigue, resulting in an increased susceptibility of younger patients.

Concomitant depressive symptoms and sleep problems were strongly associated with fatigue scores. This is not surprising, given that questionnaires for fatigue and depressive symptoms overlap in several items (e.g. loss of energy, sleep problems). Moreover, it might be hypothesised that the increase in depression and fatigue scores may be partly due to general pandemic-related stressors that are not related to the SARS-CoV-2 infection. However, longitudinal studies in Germany and elsewhere showed only small increases in depression scores and no relevant increase in anxiety scores during the first year of the pandemic compared to pre-pandemic years.^40^, ^41^ Indeed, mean anxiety scores on the GAD-7 were not higher in our patient sample than in a German general population survey during the same period.^40^ It is thus unlikely that patients in our study were substantially more distressed than the general population. Taken together, these findings suggests that the observed frequency and severity of post-COVID fatigue substantially exceed the effects of general pandemic-related psychosocial distress.

At an average of 9 months after infection, 26% of patients in our study showed mild and 1% moderate cognitive impairment. Although MoCA data from controls was not available for our analyses, cognitive impairment was about twice as common in the oldest age group as would be expected from normative data.^42^ Given that middle-aged healthy individuals tend to score well above the cut-off for cognitive impairment,^43^ our findings suggest that the frequency of post-COVID cognitive impairment is substantially increased across all age groups.

Cognitive impairment was best predicted by sociodemographic factors and pre-COVID neurological comorbidity, which is consistent with findings from a recent meta-analysis.^6^ The identified factors are well-established risk factors of cognitive impairment and dementia in the general population. Interestingly, disease severity showed no independent association in multivariable analyses. This suggests that higher age, lower socio-economic status and neurological comorbidity may be risk factors for both, more severe COVID-19 and long-term cognitive deficits. It is therefore possible that a SARS-CoV-2 infection may either exacerbate or unmask ongoing cognitive decline.

Overall, only about 5% of patients suffered from both cognitive impairment and fatigue in our study and there was no statistically significant association between the two syndromes in univariate and multivariable models. Indeed, fatigue and cognitive impairment showed distinct age distributions: while the relative frequency of fatigue was highest in patients younger than 25 years and decreased with age, the frequency of cognitive impairment increased with age. Importantly, fatigue and cognitive impairment were associated with different characteristics: While cognitive impairment was mostly associated with general sociodemographic risk factors, fatigue showed strong associations with psychiatric comorbidity and early COVID-related neurological involvement. Finally, our cross-sectional analyses show distinct temporal patterns that are consistent with earlier longitudinal studies: Fatigue appears to occur during or shortly after the acute phase and then slowly improves over time,^30^ whereas the onset of cognitive impairment or even dementia may be delayed by several months after infection.^44^, ^45^ Overall, these findings indicate that fatigue and cognitive impairment are two distinct sequelae of COVID-19 with potentially different underlying pathophysiological mechanisms. This suggests that future studies investigating the aetiology of these syndromes should address them separately and assume distinct pathophysiological mechanisms.

The population-based NAPKON cohort is representative of the background population in all major sociodemographic characteristics^15^ and the proportion of hospitalised patients in our sample (6%) parallels the hospitalization rate in Germany during the study period.^46^ Additional strengths of our study include the well-matched control group, use of validated instruments and detailed data on sociodemographic and clinical characteristics, comorbidity, blood tests and psychiatric symptoms.

Our study has the following limitations. The control group was recruited before the outbreak of the COVID-19 pandemic, i.e. the control group was not exposed to the same pandemic-related social and economic stressors. However, choosing a pre-pandemic control group ensured that none of the control participants had been infected with SARS-CoV-2, which we consider to be a strength of the study. The control group did not receive a cognitive assessment and results on post-COVID cognitive deficits therefore were interpreted relative to normative data. The response rate was lower in the patient group than in the control group, potentially due to differences in study setting (on-site vs. at home assessment). As with other studies using on-site assessments, the requirement to travel to the study centre may have prevented some patients from participating in the study. Future studies should estimate the resulting risk of bias through non-responder analyses. Detailed non-responder data were not available in our case due to the data protection policy of the public health authorities who sent out the study invitations on our behalf. In addition, the cross-sectional design limits conclusions about the trajectory of symptoms over time.

Individuals infected with SARS-CoV-2 show a statistically significantly increased rate of fatigue. Patients with a neuropsychiatric comorbidity and more acute COVID symptoms, and especially those with neurological symptoms, show particularly high rates of post-COVID fatigue. In addition, post-COVID cognitive deficits were common in all age groups. Importantly, our results indicate that fatigue and cognitive impairment are distinct sequelae of COVID-19 with separate risk factors and demographic features.

## Contributors

Tim J. Hartung was responsible for conceptualization, data curation, formal analysis, methodology, project administration, resources, software, visualisation, writing of the original draft, review and editing.

Christian Neumann was responsible for data curation, investigation, methodology, project administration, writing of the original draft, review and editing.

Thomas Bahmer was responsible for conceptualization, funding acquisition, investigation, methodology, project administration, resources, supervision, review and editing.

Irina Chaplinskaya-Sobol was responsible for data curation, project administration, resources, review and editing.

Matthias Endres was responsible for resources, supervision, review and editing.

Johanna Geritz was responsible for data curation, investigation, review and editing.

Karl Georg Haeusler was responsible for methodology, project administration, resources, supervision, review and editing.

Peter U. Heuschmann was responsible for methodology, project administration, resources, supervision, review and editing.

Hanna Hildesheim was responsible for investigation, review and editing.

Andreas Hinz was responsible for data curation, formal analysis, investigation, methodology, resources, supervision, review and editing.

Sina M. Pütz was responsible for project administration, resources, review and editing.

Anna Horn was responsible for data curation, investigation, methodology, review and editing.

Michael Krawczak was responsible for supervision, review and editing.

Lilian Krist was responsible for investigation, methodology, review and editing.

Jennifer Kudelka was responsible for data curation, investigation, review and editing.

Wolfgang Lieb was responsible review and editing.

Corina Maetzler was responsible for data curation, investigation, review and editing.

Anja Mehnert-Theuerkauf was responsible for project administration, resources, supervision, review and editing.

Felipe A. Montellano was responsible for data curation, investigation, methodology, review and editing.

Caroline Morbach was responsible for investigation, review and editing.

Sein Schmidt was responsible for project administration, resources, review and editing.

Stefan Schreiber was responsible for resources, review and editing.

Flo Steigerwald was responsible for formal analysis, methodology, software, review and editing.

Stefan Störk was responsible for review and editing.

Walter Maetzler was responsible for conceptualization, funding acquisition, methodology, project administration, resources, supervision, writing of the original draft, review and editing.

Carsten Finke was responsible for conceptualization, funding acquisition, methodology, project administration, resources, supervision, visualisation, writing of the original draft, review and editing.

Tim J. Hartung, Christian Neumann, Andreas Hinz and Flo Steigerwald have verified the underlying data.

All authors had full access to all the data in the study and accept responsibility to submit for publication.

## Data sharing statement

All data of this study may be shared upon request to the NAPKON data use and access committee. Interested parties can access relevant data governance information and submit their research proposal to the NAPKON use and access committee at https://proskive.napkon.de.

## Declaration of interests

K.G.H. reports speaker's honoraria, consulting fees, lecture honoraria and/or study grants from Abbott, Amarin, AstraZeneca, Bayer Healthcare, Biotronik, Boehringer Ingelheim, Bristol-Myers Squibb, Daiichi Sankyo, Edwards Lifesciences, Medronic, Pfizer, Portola, Premier Research, Sanofi, SUN Pharma, and W.L. Gore and Associates, all of which were unrelated to this work. ME reports grants from Bayer and fees paid to the Charité from Abbot, Amgen, AstraZeneca, Bayer, Boehringer Ingelheim, BMS, Daiichi Sankyo, GSK, Sanofi, Covidien, Novartis and Pfizer, all of which were unrelated to this work. The other authors have reported no potential conflicts of interest.

## Appendix Supplementary materials

Image, application 1
