# Supplementary material for: Fatigue and cognitive impairment after COVID-19: A prospective multicentre study
Source: eClinicalMedicine. 2022 Sep 17;53:101651. doi: 10.1016/j.eclinm.2022.101651 (PMC9482331; doi:10.1016/j.eclinm.2022.101651)
Supplement: Supplementary file 1 [file mmc1.pdf]

Online-Only Supplement

Fatigue and cognitive impairment after COVID-19: a prospective multicentre study

Tim J. Hartung, MD MA[1,2] *, Christian Neumann, BSc[3] *, Thomas Bahmer, MD[4,5], Irina Chaplinskaya-Sobol, MSc[6], Matthias Endres, MD[1,7,8,9,10], Johanna Geritz, Dipl.-Psy.[3], Karl Georg Haeusler, MD[11], Peter U. Heuschmann, MD[12,13], Hanna Hildesheim, MD[3], Andreas Hinz, PhD[2], Sina M. Pütz, MD[14], Anna Horn[12], Michael Krawczak, PhD[15], Lilian Krist, MD[16], Jennifer Kudelka, MD[3], Wolfgang Lieb, MD MSc[17], Corina Maetzler, MA[3], Anja Mehnert-Theuerkauf, PhD[2], Felipe A. Montellano, MD[12,11,18], Caroline Morbach, MD[19], Sein Schmidt, MD[20], Stefan Schreiber, MD[21], Flo Steigerwald[1], Stefan Störk, MD, PhD[22,23], Walter Maetzler, MD[3] *, Carsten Finke, MD[1] *

* contributed equally

1 Klinik und Hochschulambulanz für Neurologie, Charité-Universitätsmedizin Berlin
2 Department of Medical Psychology and Medical Sociology, University Medical Center Leipzig, Leipzig, Germany
3 Neurology Department, University Medical Center Schleswig-Holstein, Campus Kiel
4 Internal Medicine Department I, University Hospital Schleswig Holstein, Campus Kiel
5 Airway Research Center North (ARCN), German Center for Lung Research (DZL)
6 Department of Medical Informatics, University Medical Center Göttingen
7 Center for Stroke Research Berlin
8 Excellence Cluster NeuroCure
9 German Center for Neurodegenerative Diseases (DZNE), partner site Berlin
10 German Centre for Cardiovascular Research (DZHK), partner site Berlin
11 Department of Neurology, Universitätsklinikum Würzburg
12 Institute of Clinical Epidemiology and Biometry, University of Würzburg
13 Clinical Trial Center, University Hospital Würzburg
14 University of Cologne, Faculty of Medicine and University Hospital Cologne, Department I of Internal Medicine, Center for Integrated Oncology Aachen Bonn Cologne Duesseldorf
15 Institute of Medical Informatics and Statistics, Kiel University, University Medical Center Schleswig-Holstein Campus Kiel
16 Institute of Social Medicine, Epidemiology and Health Economics, Charité-Universitätsmedizin Berlin
17 Institute of Epidemiology, Kiel University, Kiel, Germany
18 Comprehensive Heart Failure Center, University and University Hospital Würzburg
19 University Hospital Würzburg, Department for Medicine I and Comprehensive Heart Failure Center
20 Berlin Institute of Health at Charité – Universitätsmedizin Berlin, Clinical Study Center
21 Universitätsklinikum Schleswig-Holstein, Christian-Albrechts-Universität zu Kiel, Klinik für Innere Medizin I
22 Department of Clinical Research & Epidemiology, Comprehensive Heart Failure Center, University Hospital Würzburg
23 Department of Internal Medicine I, University Hospital Würzburg

eText 1. Supplementary Acknowledgements.

We thank all representatives of contributing NAPKON sites: University Hospital Schleswig-Holstein, Kiel (Enderle J), University Hospital Wuerzburg, Wuerzburg (Frantz S), University Hospital Wuerzburg, Wuerzburg (Hein G), University Hospital Schleswig-Holstein, Kiel (Hermes A), University Hospital Wuerzburg, Wuerzburg (Isberner N), University Hospital Wuerzburg, Wuerzburg (Jahns R), University Hospital Wuerzburg, Wuerzburg (Kohls M), University Hospital Schleswig-Holstein, Kiel (Lehmann I), University Hospital Schleswig-Holstein, University Hospital Wuerzburg, Wuerzburg (Reese J-P), Kiel (Reinke L), University Hospital Schleswig-Holstein, Kiel (Tittman L), University Hospital Wuerzburg, Wuerzburg (Weissbrich B).

We also thank all representatives of participating NUM infrastructure that contributed to this study: University Hospital Frankfurt, Frankfurt (Appel K), University Medicine Greifswald, Greifswald (Bahls T), University of Wuerzburg, Wuerzburg (Bauer C), University Hospital Cologne, Cologne (Brechtel M), University Medicine Goettingen, Goettingen (Chaplinskaya I), University of Wuerzburg, Wuerzburg (Fiessler C), University Hospital Cologne, Cologne (Fuhrmann S), University of Wuerzburg, Wuerzburg (Goester M), University of Wuerzburg, Wuerzburg (Grau A), University Medicine Goettingen, Goettingen (Hanß S), German Center for Cardiovascular Diseases (DZHK), Berlin (Hoffmann J), University Medicine Greifswald, Greifswald (Hoffmann W), University Hospital Cologne, Cologne (Pütz SM), University of Wuerzburg, Wuerzburg (Jiru-Hillmann S), University of Wuerzburg, Wuerzburg (Kammerer K), University of Wuerzburg, Wuerzburg (Kohls M), Helmholtz Center Munich, Munich (Kraus M), University Medicine Goettingen, Goettingen (Krefting D), University Hospital Cologne, Cologne (Lee C), Charité - Universitaetsmedizin Berlin, Berlin (Lorbeer R), University of Wuerzburg, Wuerzburg (Miljukov O), University Medicine Greifswald, Greifswald (Nauck M), University Hospital Cologne, Cologne (Nunnendorf M), University Medicine Goettingen, Goettingen (Pape C), University Medicine Greifswald, Greifswald (Schäfer C), Charité - Universitaetsmedizin Berlin, Berlin (Schaller J), University Medicine Greifswald, Greifswald (Schattschneider M), University Hospital Cologne, Cologne (Schmidt Ibanez C), University Hospital Frankfurt, Frankfurt (Schneider J), University Medicine Greifswald, Greifswald (Stahl D), University of Wuerzburg, Wuerzburg (Ungethuem K), University Medicine Greifswald, Greifswald (Valtentin H), University Hospital Cologne and University Hospital Frankfurt, Cologne and Frankfurt (Vehreschild J).

In addition, we are grateful to the NAPKON Steering Committee: University Hospital Giessen and Marburg, Giessen (Herold S), University of Wuerzburg, Wuerzburg (Heuschmann P), Charité - Universitaetsmedizin Berlin, Berlin (Heyder R), University Medicine Greifswald, Greifswald (Hoffmann W), Hannover Unified Biobank, Hannover Medical School, Hannover (Illig T), Robert Koch Institute, Department of Epidemiology and Health Monitoring, Berlin (Neuhauser H), University Hospital Schleswig-Holstein, Kiel (Schreiber S), University Hospital Cologne and University Hospital Frankfurt, Cologne and Frankfurt (Vehreschild J), Jena University Hospital, Jena (von Lilienfeld-Toal M), Charité - Universitaetsmedizin Berlin, Berlin (Witzenrath M).

And we thank the NAPKON Use & Access Committee: University Medicine Goettingen, Goettingen (Blaschke S), Lahn-Dill-Clinics, Wetzlar (Ellert C), LMU clinic of the university of munich, Munich (Frank S), University Hospital Schleswig-Holstein, Kiel (Friedrichs A), University of Wuerzburg, Wuerzburg (Heuschmann P), Hannover Unified Biobank, Hannover Medical School, Hannover (Illig T), University Hospital Wuerzburg, Wuerzburg (Meybohm P), LMU clinic of the university of munich, Munich (Milger K), University

Medicine Oldenburg, Oldenburg (Petersmann A), University Hospital Technical University Munich , Munich (Schmidt G), University Hospital Schleswig-Holstein, Kiel (Schreiber S), University Hospital Cologne and University Hospital Frankfurt, Cologne and Frankfurt (Vehreschild J), Jena University Hospital, Jena (von Lilienfeld-Toal M), Charité - Universitaetsmedizin Berlin, Berlin (Witzenrath M), University Hospital Essen, Essen (Witzke O).

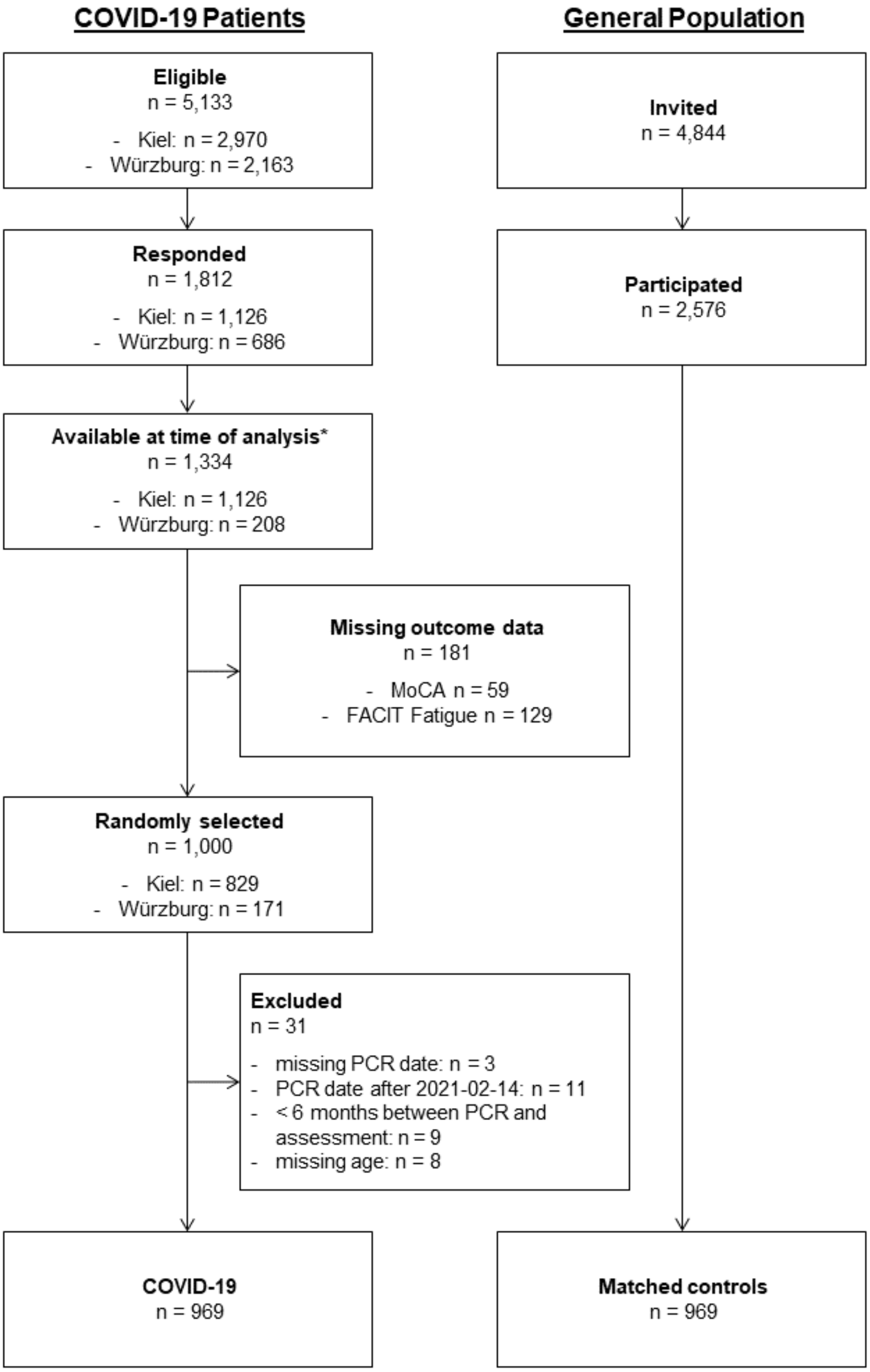

eFigure 1. Recruitment flow chart. Data collection and recruitment are still ongoing for this cohort. The required sample size of n=1000 was based upon previous power calculations. The final data set was compiled as soon as the sample size was reached. * At the time of data compilation (19 January 2022).

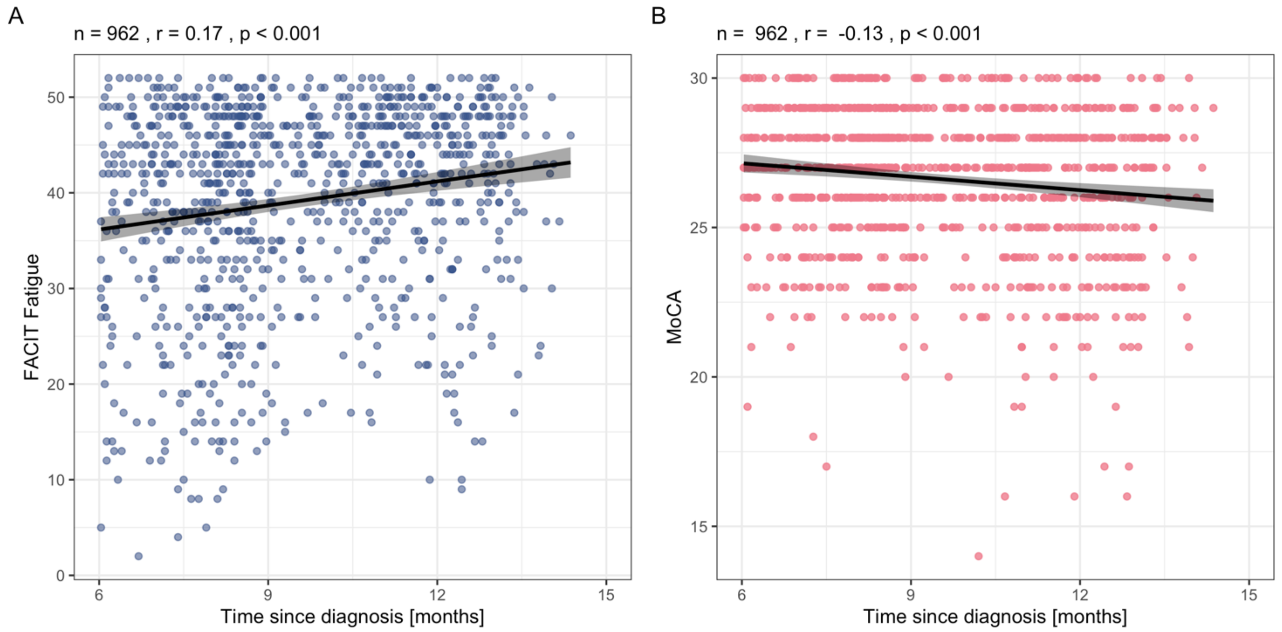

eFigure 2. Sensitivity analysis excluding n = 7 patients assessed > 15 months after infection: Association between time since diagnosis of SARS-CoV-2 infection and (A) FACIT fatigue score and (B) Montreal Cognitive Assessment (MoCA) score. r, Pearson correlation coefficient, trend line indicates univariate linear regression, shaded area 95% confidence interval.

eTable 1. Detailed patient sample characteristics and main outcomes including number of available cases (N) for each variable.

Characteristic	N	n (%) / M (SD)
**Pre-COVID comorbidity**		
Migraine	958	92 (10%)
Epilepsy	966	9 (1%)
Ischemic stroke	965	8 (1%)
Multiple sclerosis	969	5 (1%)
Parkinson	969	1 (0%)
Haemorrhagic stroke	964	0 (0%)
Dementia	968	0 (0%)
Other neurological disease	967	6 (1%)
Depression disorder	956	100 (10%)
Anxiety disorder	965	33 (3%)
Psychotic disorder	967	3 (0%)
Hypertension	966	222 (23%)
Coronary artery disease	962	33 (3%)
Atrial fibrillation	767	17 (2%)
Myocardial infarction	966	11 (1%)
Other arrhythmia	767	10 (1%)
Heart failure	955	9 (1%)
Carotid artery stenosis	765	3 (0%)
Peripheral artery disease	766	0 (0%)
Other cardiovascular disease	954	62 (6%)
Long-term medication	921	488 (53%)
**Clinical characteristics**		
Covid wave	969	
First		481 (50%)
Second		488 (50%)
Hospitalization duration [days]	63	32 (86)
**Acute Covid Symptoms**		
Headache	882	662 (75%)
Limb pain	887	655 (74%)
Dysgeusia	884	611 (69%)
Dysosmia	871	602 (69%)
Cough	890	589 (66%)
Sore throat	882	568 (64%)
Fever	871	549 (63%)
Myalgia	872	513 (59%)
Dyspnoea	873	506 (58%)
Chills	889	425 (48%)
Rhinorrhoea	871	416 (48%)
Dizziness	869	393 (45%)
Thorax pain	877	286 (33%)
Hoarseness	873	248 (28%)
Diarrhoea	873	221 (25%)
Nausea	876	204 (23%)
Wheeze	859	203 (24%)
Confusion	856	152 (18%)
Abdominal pain	872	151 (17%)
Hair loss	770	87 (11%)
Rash	885	80 (9%)
Vomiting	901	41 (5%)
Other acute COVID symptoms	897	353 (39%)

**Post-COVID characteristics**		
Any persisting symptoms	896	473 (53%)
Anaemia	958	74 (8%)
Macrocytic hyperchromic		2 (3%)
Microcytic hypochromic		18 (24%)
Normocytic normochromic		54 (73%)
Thyroid hormones	923	
Latent hyperthyroidism		1 (0%)
Latent hypothyroidism		26 (3%)
Low T3 syndrome		58 (6%)
Normal		838 (91%)
MoCA (M, SD)	969	27 (2)
Cognitive impairment	969	
Mild impairment		256 (26%)
Moderate impairment		7 (1%)
No impairment		706 (73%)
FACIT fatigue (M, SD)	969	39 (10)
Fatigue severity	969	
Normal		423 (44%)
Mild (≤43)		358 (37%)
Clinically relevant (≤30)		188 (19%)
Depression symptom severity (PHQ-8)	946	
None/minimal		523 (55%)
Mild		273 (29%)
Moderate		103 (11%)
Moderately Severe		38 (4%)
Severe		9 (1%)
Anxiety symptom severity (GAD-7)	948	
None/minimal		685 (72%)
Mild		180 (19%)
Moderate		59 (6%)
Severe		24 (3%)
Sleep problems (PSQI ≥5)	634	513 (81%)

eTable 2. Multivariable linear regression model of acute COVID symptoms associated with FACIT fatigue, ≥6 months after infection (R^2=0.19).

**Characteristic**	**Beta**	**95% CI[1]**	**p**
Altered consciousness	-4.1	-6.1, -2.1	**<0.001**
Dizziness	-3.1	-4.7, -1.6	**<0.001**
Myalgia	-2.7	-4.2, -1.1	**<0.001**
Thorax pain	-2.5	-4.1, -0.89	**0.002**
Dyspnoea	-2.0	-3.5, -0.48	**0.010**
Dysosmia	-1.6	-3.1, -0.14	**0.033**
Rash	-2.4	-4.8, -0.07	**0.044**
